# Speciation, Divergence, and the Origin of *Gryllus rubens*: Behavior, Morphology, and Molecules

**DOI:** 10.3390/insects2020195

**Published:** 2011-05-04

**Authors:** David A. Gray

**Affiliations:** Department of Biology, California State University, Northridge, 18111 Nordhoff Street, Northridge, CA 91330, USA; E-Mail: dave.gray@csun.edu

**Keywords:** speciation, crickets, behavioral isolation, phylogeography

## Abstract

The last 25 years or so has seen a huge resurgence of interest in speciation research. This has coincided with the development and widespread use of new tools in molecular genetics, especially DNA sequencing, to inform ecological and evolutionary questions. Here I review about a decade of work on the sister species of field crickets *Gryllus texensis* and *G. rubens*. This work has included analysis of morphology, behavior, and the mitochondrial DNA molecule. The molecular work in particular has dramatically re-shaped my interpretation of the speciational history of these taxa, suggesting that rather than ‘sister’ species we should consider these taxa as ‘mother-daughter’ species with *G. rubens* derived from within a subset of ancestral *G. texensis*.

## 1. Introduction

The process of speciation remains one the central issues in evolutionary biology. For most of the *ca.* 150 years since Darwin's *Origin*, speciation research was lower profile, and lower priority, than other major topics in evolution, namely (1) the integration of Mendelian genetics and the flourishing of quantitative population genetics, and (2) the measurement of allozyme genetic variation in wild populations. Evolution by natural selection (inclusive of sexual selection) was generally considered to be such a slow process that intrinsically historical research agendas, such as speciation, were considered nearly intractable. Subsequent direct observation of natural selection in the wild [1] as well as renewed interest in sympatric speciation, starting most prominently with Guy Bush's work with *Rhagoletis* [2], have together revitalized interest in speciation research and made it one of the most active areas within evolutionary biology today [3–10]. At about the same time, there have been enormous advances in molecular genetics, particularly DNA sequencing, as well as bioinformatics approaches to phylogenetics and phylogeography [11]. Today it would be almost inconceivable to study speciation without molecular data bolstering interpretations of host shifts [12,13], sympatry [14,15], and/or adaptive genetic divergence [16,17]. In this review, I will re-cap about a decade of work on speciation and reproductive isolation in the southern USA trilling field crickets, *Gryllus rubens* and *Gryllus texensis*. Rather than follow this research strictly chronologically, I will move from behavior, to eco-morphology, to molecular genetics, while hopefully illustrating the singularly enormous contribution that the molecular genetic data has made to my current thinking about speciation of these taxa. I will also indulge in far more speculation than is typically allowed in publications of primary scientific data sets. Hopefully many of those speculations will one day be tested, whether shown to be correct or not. More specifically, I hope that other researchers, including graduate students and postdocs (especially those savvy with advanced molecular techniques) find this system worthy of their time and study.

## 2. Reproductive Behavior

Acoustically communicating insects have been among the many ‘non-model’ models of speciation research over the past 40 or so years. Because of the prominence Dobzhansky and Mayr gave allopatric divergence followed by the reinforcement of pre-zygotic barriers as a model of speciation [18,19]; reproductive character displacement, that is, greater separation of species-specific reproductive characters in sympatry than in allopatry, became a key element in the search for indirect evidence of the process of speciation [20]. One of the most influential of the early reviews of reproductive character displacement compiled data from acoustically communicating insects, primarily crickets and katydids, and concluded that the evidence for reproductive character displacement was scarce at best [21]. Although there are now several strong examples of reproductive character displacement in acoustically communicating insects [22,23], it was against the background of Walker's influential review that Bill Cade and I undertook to test for behavioral divergence and reproductive character displacement between the southeastern USA trilling field crickets *Gryllus rubens* and *G. texensis* (for photos and songs, see the Singing Insects of North America website [24]). What we hoped would make our study significant was our ability to test female responses to male song in addition to male song parameters, in particular pulse rate. We decided to do this using the first generation offspring of wild caught females, isolated from males after capture, which would therefore be sets of full and/or half siblings with paternity that reflected female mating decisions in the wild.

**Figure 1:**
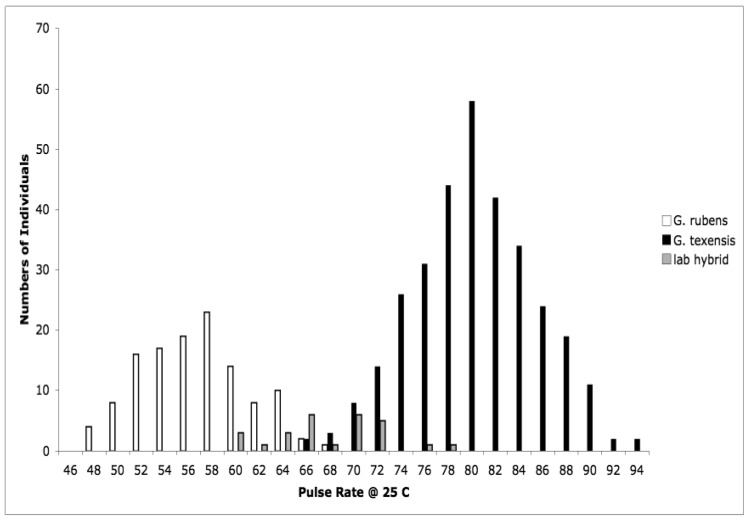
Pulse rates in the calling songs of males (corrected to a common temperature) are strongly bimodal and show almost no overlap; laboratory produced hybrids are intermediate.

Detailed methodology is provided in [25], but basically what we did was collect adult females from throughout the range of *G. texensis*, bring those females into the lab, and rear each female's offspring separately. After adult eclosion we recorded the calling songs of males, and we tested females' responses to variation in song. Thus for each wild-caught female we obtained pulse rate data from her sons, and female preference for pulse rate data from her daughters. From these data, for *G. texensis*, we were able to show (1) that male pulse rate has heritable genetic variation, with *h^2^* roughly 40%, (2) that female preference for pulse rate also has heritable genetic variation, *ca.* 38%, (3) that there was a significant genetic correlation between male pulse rate and female preference for pulse rate, *r_G_* = 0.49, and (4) that neither male song nor female preference for song differed across the geographic range in a way consistent with reproductive character displacement—there was in fact strikingly little geographic variation in *G. texensis* song and preference. At the time we did not collect enough *G. rubens* to conduct a parallel test in that species, but subsequent work has shown that *G. rubens* also lacks reproductive character displacement in male calling song [26] and that extensive field collections show strongly bimodal nearly non-overlapping pulse rates (Figure 1).

We interpreted those results in the context of the predictions of the reinforcement model, and in light of what we already knew about hybrid viability in crosses of *G. texensis* and *G. rubens*. The essence of the reinforcement model of speciation is that selection acts against hybrid mating because of the lower fitness of hybrids, which in turn is due to divergence that has already taken place in allopatry. That is, the reinforcement model specifically predicts that hybrids are unfit—*i.e.*, some degree of post-zygotic isolation exists, which therefore selects for increased pre-zygotic isolation. From previous work with these species, we already knew that viable hybrids could be formed in the laboratory [27,28], and that they had pulse rates intermediate between *G. rubens* and *G. texensis* [29,30], see Figure 1. So it appeared to us that neither the preconditions of the reinforcement model, post-zygotic isolation, nor its predictions, reproductive character displacement, were met. Instead we interpreted our results as suggesting the primary importance of sexual selection causing song divergence—and thereby effecting reproductive isolation and speciation. The sexual selection model was indirectly supported by the female preference data, and by the quantitative genetic data including a genetic correlation sufficiently strong to suggest the potential for rapid Fisherian runaway coevolution of male trait and female preference [31,32].

Our studies of cricket reproductive isolation had thus far mostly emphasized the long distance and comparatively loud calling song. It is the pulse rate of that song that we had determined was the principal character that we could use to delimit the species, and it also seems to be of primary importance for how the crickets themselves recognize species. However cricket mating behavior is considerably more complex than just female phonotaxis to male calling song; we thought it was important to characterize the close-range courtship interactions between males and females to gain a more complete picture of reproductive isolation. In field crickets generally, the mating sequence could be characterized as (1) male calling; (2) female phonotaxis to calling song; (3) mutual antennation and assessment of cuticular hydrocarbons; (4) male courtship song; (5) spermatophore transfer, and (6) sperm storage and utilization [33–35]. Steps 1 and 2, male calling song and female phonotaxis, do not always precede mating however. Many males call infrequently or not at all [36-38], and may adopt a satellite strategy [39,40], and/or actively search for females especially at high population density [41–43]. Reduced male calling activity may reflect a history of attack by the tachinid fly, *Ormia ochracea*, which locates hosts via the male's calling song [44–49].

**Figure 2:**
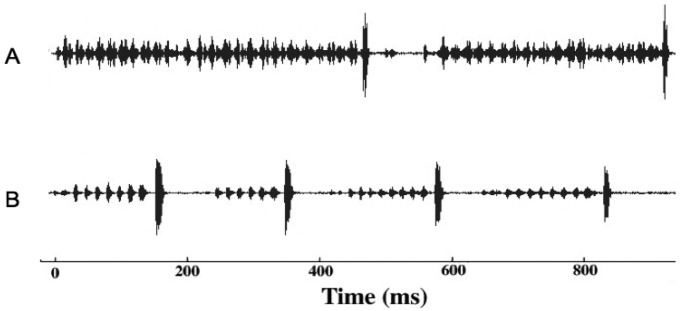
Waveform (amplitude *versus* time) representative courtship songs of *G. rubens* (Figure 2a) and *G. texensis* (Figure 2b).

**Figure 3:**
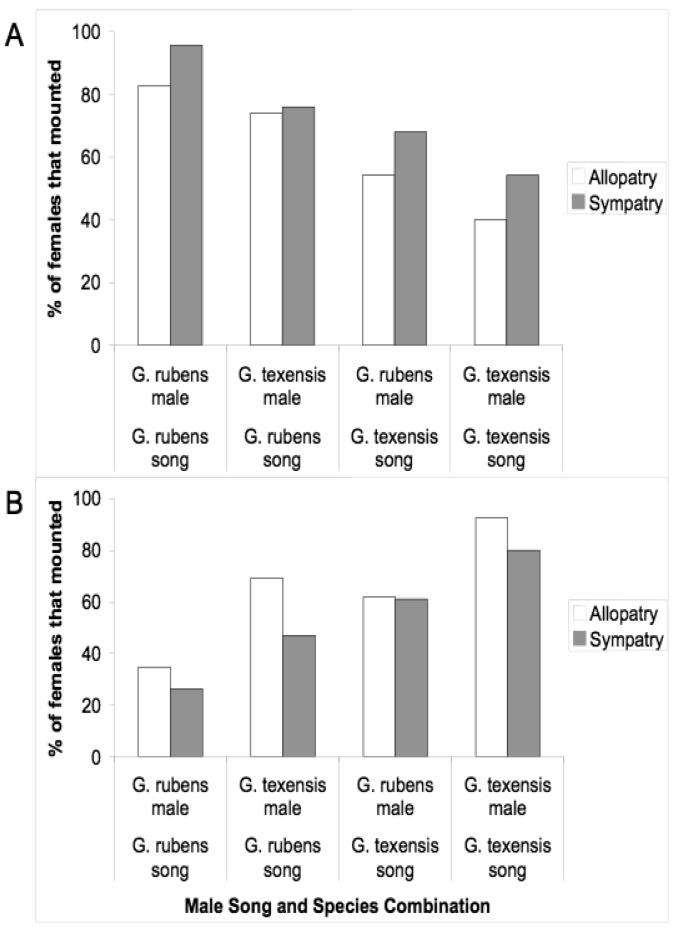
Rates of female acceptance of male courtship when courtship song is experimentally decoupled from male species identity; muted males were accompanied by the species-average courtship song indicated. There were separate statistically significant effects of both male species identity and courtship song played, but no significant effect of female allopatry or sympatry for both *G. rubens* females (Figure 3a) and *G. texensis* females (Figure 3b). Figure modified from Gray (2005).

Given that males and females may encounter one another without calling song, we conducted several studies to examine divergence in male courtship song, and in male and female close-range mating behaviors. Courtship song in *G. rubens* and *G. texensis* is composed of a series of quieter mostly pure tone lower-frequency ticks (*ca.* 5 kHz) with louder broader spectrum higher-frequency ticks (most sound energy 11–14 kHz) at regular intervals (Figure 2). Just by recording and analyzing male courtship songs, we were able to show that *G. rubens* and *G. texensis* differ in their courtship song rates in a manner that mirrors their calling song pulse rates: *G. texensis* has faster courtship song than *G. rubens* [50], and experimental manipulation of the diet showed that courtship song features did not appear to reflect male nutritional condition [51]. Based on this, my lab conducted two separate studies of close-range courtship and mating behavior with these species. The first [52], used muted males with virgin females of both species. Males that courted females, although themselves mute, were accompanied by a synthetic species average courtship song played via a tweeter directly under the pair of crickets. Thus males had the opportunity to court females, or not, and females had two experimentally separated sources of information about male species identity (1) from their cuticular hydrocarbons (unmanipulated) and (2) from the accompanying courtship song (manipulated). I replicated the entire experiment with crickets from allopatry and sympatry, using *G. texensis* from Austin, Texas and Tuscalloosa, Alabama, and *G. rubens* from Gainesville, Florida, and from Tuscallosa, Alabama. In short, the results showed separate significant effects of male species identity and courtship song played on the likelihood of female mounting; there was no difference in either species between the behaviors of crickets from sympatry and allopatry (Figure 3). Although these results show divergence in close-range courtship mating behaviors, they also notably show that despite significant species effects, the potential for hybridization is not trivial—on the assumption of no prior female phonotaxis to male calling song.

**Figure 4:**
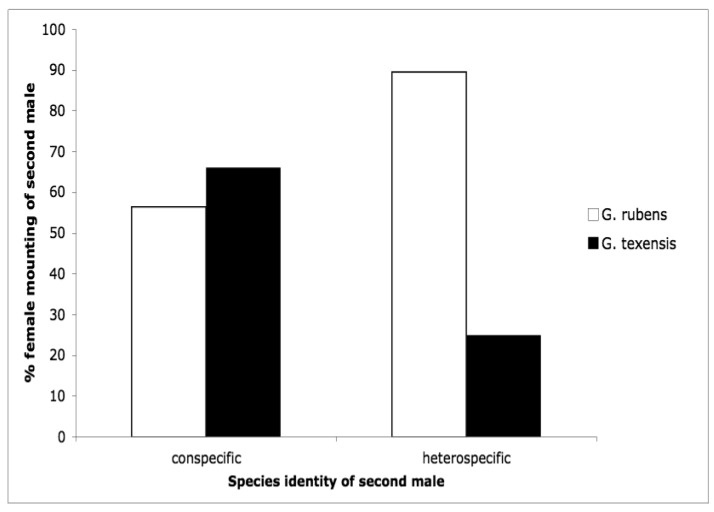
Female responsiveness to males in sequential mating trials in which the first male was a conspecific, and the second male was either a conspecific or heterospecific.

The second courtship study we conducted was motivated by the idea that females may control paternity via selectively re-mating. Female crickets generally are polyandrous [53–63] and *G. texensis* is no exception [64]. Because sperm competition in these crickets favors the last male to mate (average paternity of the second male to mate in a study of *G. texensis* was 72% [65]), females first mated to a conspecific should be very reluctant to engage in a second mating with a heterospecific. Our results somewhat supported these predictions [66], but differed in some interesting and suggestive ways. Males of both species courted females of both species at equal rates [66], as I had found for *G. texensis* males in my previous study [52]; *G. rubens* males had preferentially courted conspecifics in that study. *G. texensis* females basically met our predictions: upon first exposure to males, female *G. texensis* preferred conspecific males, and they preferred conspecific males following exposure to conspecific males, *i.e.*, they were unwilling to ‘trade-down’ to heterospecific males. Choosiness was relaxed following first exposure to heterospecific males. Female *G. rubens*, however, appeared to prefer heterospecific males upon either first or subsequent exposure. Results for both species are summarized in Figure 4. The lack of choosiness in *G. rubens* we interpreted in light of a Kaneshiro effect and what we then knew about their evolutionary history (see below).

To summarize the results discussed so far: behavioral reproductive isolation between these taxa appears to be very strong. First, isolation by male calling song and female phonotaxis approaches 100%—male pulse rate distributions are nearly non-overlapping, and females show strong preferences for pulse rates of conspecifics. Second, in those few instances of phonotaxis to heterospecific song that might occur [or encounters in the absence of calling song (*i.e.*, via random encounter)] courtship interactions reduce the likelihood of mating, mostly mediated by female selectivity not male selectivity. These studies, while informative, obviously beg the question of eco-morphological divergence, and the degree of genetic isolation and divergence. That is, the observed levels of species behavioral divergence, the absence of readily identifiable hybrids in the field, and the results showing strong behavioral isolation all suggest that some degree of ecological divergence might facilitate species persistence and also suggests that genetic reproductive isolation should be quite strong.

## 3. Eco-Morphological Divergence

**Figure 5:**
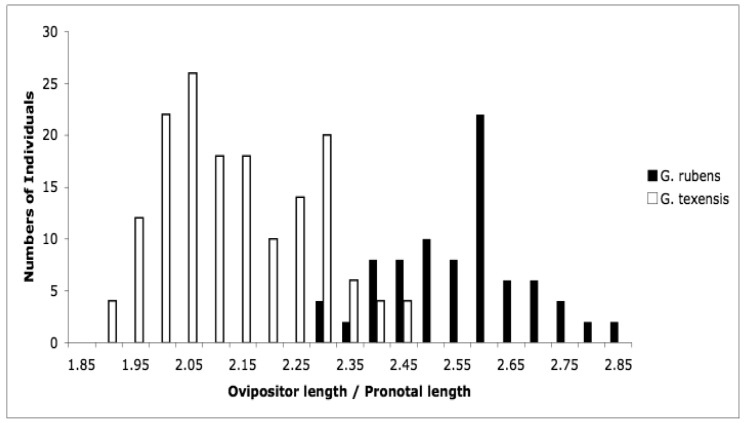
Ovipositor length relative to body size (pronotal width) in females.

At the time that Bill Cade and I started our character displacement study, there were no known morphological means of distinguishing between these two species. That situation is unchanged for males, however during our work we observed that female *G. rubens* appeared to have longer ovipositors relative to body size than did female *G. texensis*. To confirm this, we measured ovipositor length and pronotal width, and we found that females were in fact quite strongly divergent in relative ovipositor length [67]. In particular, if we make a histogram of ovipositor length divided by pronotal width, we see imperfect but bimodal separation (Figure 5). Ovipositor length in crickets is clearly an ecologically linked trait; effects of phylogenetic history, latitude, egg size, oviposition behavior, diapause strategy, and soil type have all been described [68–73]. *G. rubens* and *G. texensis* share their deeper phylogenetic history, are found at similar latitudes, and both have facultative nymphal diapause with no egg diapause observed. Egg size, oviposition strategy, and soil preferences have not been examined, but *G. rubens*, in Florida and the southeastern coastal areas especially, experiences sandier soils than elsewhere throughout the species' ranges. Sandy soils drain and dry quickly compared to more loamy or clay-laden soils, and crickets are known to oviposit deeper in dryer sandy soil [73]. The congeneric species pair, *G. firmus* (known as the ‘sand field cricket’) and *G. pennsylvanicus* show a similar pattern: both have recent shared evolutionary history, both are egg diapausing species, but *G. firmus* has a considerably longer ovipositor than the more inland *G. pennsylvanicus* [71,74]. Ovipositor length thus appears to be an indicator of adaptive ecological divergence between *G. rubens* and *G. texensis*. This would be especially true for *G. rubens* populations in the southeastern US coastal areas, including peninsular Florida.

## 4. Genetic Divergence and Species History

Unfortunately, at the present time, the only data available to test genetic divergence and illuminate the species' deeper evolutionary history are mitochondrial sequence data; however those mitochondrial data show clear and interesting patterns that greatly inform our understanding of speciation in these taxa.

Our first study was with the mitochondrial Cytochrome c Oxidase I (COI) and cytochrome b (CYT-B) genes. It showed that *G. texensis* had much higher levels of genetic variation than did *G. rubens*, that the gene trees showed species level separation, but were not reciprocally monophyletic, and that *G. rubens* appeared closely related to only one subset of the *G. texensis* variation [75]. That study consisted of only 10 individuals for each species, but the essential results were all later confirmed in a much larger analysis (see below) and our genetic variation results were concordant with previous work showing low levels of genetic variation in *G. rubens* [76]. Although informative, this first data set was nonetheless insufficient for large scale phylogeographic analysis.

**Figure 6:**
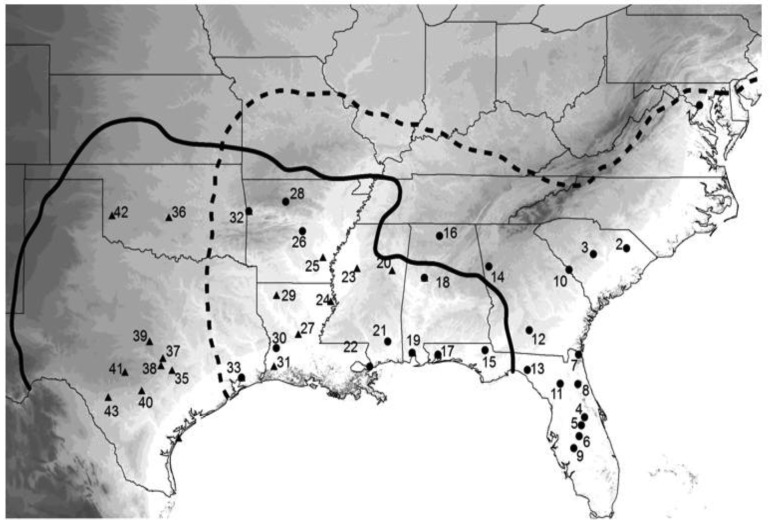
Map of the south-eastern United States of America showing collection localities for 177 *G. rubens* and 188 *G. texensis* sampled for mitochondrial DNA genetic variation. Figure modified from Gray *et al.* (2008).

To really get insight into these species' evolutionary past, I wanted a much larger sample of crickets, both in terms of numbers of individuals and geographic coverage. Fortunately, I had kept, preserved in 100% ethanol, nearly all of the many hundreds of wild caught crickets described in the previous behavioral section of this review, and had even collected or acquired a number of additional samples from parts of the species' ranges not before sampled. Because our first study [75] showed higher variation in COI than in CYT-B, I extracted DNA and amplified a portion of COI resulting in 724 aligned bases from 365 individuals from 48 collection localities spread throughout the species' ranges (Figure 6). At this point I really needed expert help with the analysis, and I was very fortunate that Lacey Knowles and Huateng Huang agreed to a collaboration. Their analysis and simulations, although in agreement with our prior results, added novel insights and completely changed how I view the evolutionary history of these two cricket species. The major results of that study [77] were that (1) *G. rubens* had far lower genetic variation than *G. texensis*, (2) *G. texensis* sequences could be divided into two mostly discrete groups, (3) *G. rubens* sequences were mostly nested within one subset of the *G. texensis* sequences, (4) that *G. rubens*, but not *G. texensis*, showed indications of both recent population expansion and isolation by distance, and (5) that recent hybridization/gene flow is an unlikely explanation for the observed lack of reciprocal gene tree monophyly.

Taken together these results suggest that *G. rubens* arose from within a subgroup of *G. texensis*, *i.e.*, that *G. rubens* is the daughter species of *G. texensis*, and that *G. rubens* arose in isolation, probably in sandy soil habitat within and/or near peninsular Florida, and has subsequently expanded demographically and geographically throughout its current range. Additionally, it seems likely that the significant song evolution that is the primary reproductive isolating barrier between these species took place in proto-*rubens* with perhaps little or no song change in *G. texensis*. Such a scenario also implies that *G. rubens* may have persisted at low population density prior to expansion. If true, this may have favored the reduced mating selectivity in *G. rubens* (compared to *G. texensis*) that we have observed in our courtship studies [66], analogous to a Kaneshiro effect [78].

**Figure 7:**
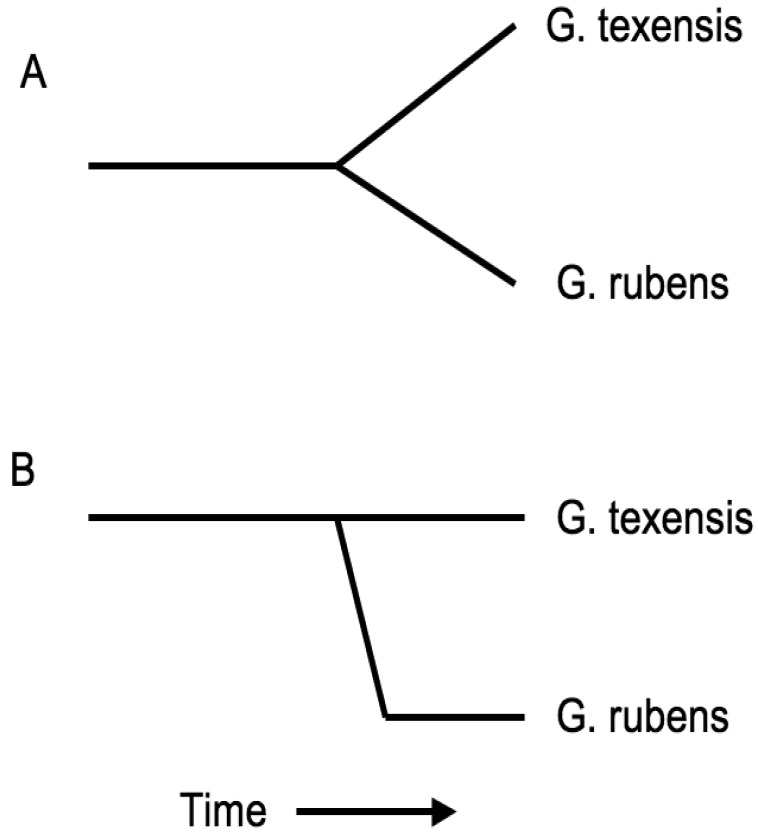
Contrasting models of speciation. Figure 7a shows the classical gradualist model with divergence attributable to approximately equal changes in both sister lineages; Figure 7b shows a punctuational view of species formation, with the majority of divergence attributable to rapid change within one ‘daughter’ lineage and relatively little change within the ‘parental’ lineage. Currently available molecular data favor a peripatric ‘puctuational’ origin of *G. rubens*.

In short, the molecular phylogeographic work [77] altered my interpretation of speciation in these taxa from a symmetric gradualist model to an asymmetric punctuational model, with the important changes being within the proto*-rubens* lineage (Figure 7). Does that imply that sexual selection and the observed genetic correlation between male song and female preference in *G. texensis* was not important in speciation? Genetic correlations can arise via pleiotropy or linkage, and in the case of sexually selected traits/preferences assortative mating can create and maintain a genetic correlation. For example, our previous work with a different *G. texensis* song character, numbers of pulses per trill (PPT), showed that there is a strong genetic correlation between male PPT and female preference for PPT in the wild, but that that genetic correlation disappears after random mating in the laboratory [79]. This demonstrates that it is assortative mating in the wild that creates and maintains the observed genetic correlation between these traits in *G. texensis*. In contrast, an important recent study of pulse rate and pulse rate preference quantitative trait loci in the Hawaiian crickets *Laupala kohalensis* and *L. paranigra* demonstrated that the observed strong genetic correlation was due mostly to either close physical linkage or pleiotropy [80]. The genetic basis of the *G. texensis* pulse rate/preference genetic correlation is unknown, and the corresponding genetic correlation has not been investigated in *G. rubens*. However, the phylogeographic data polarize the ancestral state of proto-*rubens* and suggest that that ancestor was, in effect, the *G. texensis* lineage that has led to the current *G. texensis*. As *G. texensis* of today show a strong pulse rate/preference genetic correlation, that shared ancestor likely did also, and so—in the absence of any supporting data whatsoever—it is reasonable to think that a similar genetic correlation *may* have been important in proto-*rubens* song/preference evolution.

## 5. Conclusion and Prospects for Future Work

The molecular genetic data to date have dramatically changed my interpretation of speciation between these taxa. Yet the genetic data presently available are miniscule compared to the numbers of unresolved questions that could be addressed with molecular genetics. First, and most obviously, nuclear DNA sequences, microsatellites, and/or AFLPs, *etc.* should be used to confirm or refute the major conclusions of the mitochondrial data. Second, determination of a pulse rate song/preference genetic correlation and its mechanistic basis in *G. rubens* is essential. Third, were candidate genes available, comparative work on the genetics of species divergence would be super interesting given that: (1) three features of *G. rubens* are currently known to be strongly divergent from *G. texensis:* male calling song pulse rate, female preference for calling song pulse rate, and female ovipositor length; and (2) other features are less divergent, but clearly have diverged: male courtship song rate, female preference for courtship song rate, and cuticular hydrocarbons (by implication from the courtship data in Gray 2005). For example, a comparative study of selection at the molecular genetic level for ovipositor length and cuticular hydrocarbon genes would be very interesting; I would guess that *G. rubens* ovipositor length evolved by comparatively recent strong selection and hydrocarbons by weak selection or drift; I would also guess that the courtship song and preference divergence evolved as a correlated response to the calling song divergence, probably because of pleiotropy of calling song/courtship song genes and also pleiotropy of calling song preference/courtship song preference genes. All of this would make excellent further study, and clarify the mechanistic basis of speciation. I hope someone takes it up.
